# The differences between gonadal and extra-gonadal malignant teratomas in both genders and the effects of chemotherapy

**DOI:** 10.1186/s12885-019-5598-0

**Published:** 2019-04-30

**Authors:** Hang Sun, Hongxin Ding, Jianjun Wang, Emma Zhang, Yihua Fang, Zhenhua Li, Xiao Yu, Chongren Wang, Yifan Zhao, Kan Chen, Siwan Wen, Liang Li, Shan Shan, Liu Hong, Face Chen, Pu Su

**Affiliations:** 1 Department of Endocrinology, Shanghai Tenth Peoples’ Hospital, Tongji University, No.301 Middle Yanchang Road, Shanghai, 200072 China; 2 Hospital of Yantai University, Yantai University, Yantai, China; 3 Tongji Hospital, Tongji University, Shanghai, China; 4 Mannjo Informatics Studio, Shanghai, China; 5 The First Affiliated Hospital of Anhui Medical University, Hefei, China; 6 Yantai Yuhuangding Hospital, Qingdao University School of Medicine, Yantai, China; 7 Huangshan First Peoples’ Hospital, Huangshan, China; 8 Shanghai General Hospital, Shanghai Jiaotong University School of Medicine, Shanghai, China; 9 Shanghai Sixth Peoples’ Hospital, Shanghai Jiaotong University School of Medicine, Shanghai, China; 10 Sam M. Walton College of Business, University of Arkansas, Fayetteville, AR USA; 11 Department of Informatics, Discovery & Analytical Solutions, PerkinElmer, Shanghai, No.1670, Zhang Heng Road, Zhangjiang Hi-Tech Park, Shanghai, 201203 China

**Keywords:** Malignant teratoma, Gonadal, Extragonadal, Chemotherapy

## Abstract

**Background:**

A tumor comprising of different types of tissues (such as hair, muscle, bone, etc.) is known as a teratoma. It is a type of germ cell (cells that make sperm or eggs) tumor. When these germ cells have rapid cancerous growth, then such a teratoma is called a malignant teratoma. We have studied the differences between gonadal and extra-gonadal malignant teratomas and the effects of chemotherapy in both genders.

**Methods:**

The samples of 3799 male and 1832 female patients with malignant teratoma samples, between the ages of 1 and 85+ years, were selected from the years 1973 to 2014. Trends in incidence, estimated prevalence, incidence rates, and frequency were calculated in gonadal and extra-gonadal tumors with age adjustment. The five-year observed, expected, and relative survival rates were analyzed to study the prognosis.

**Results:**

The gonadal took over a majority percentage of malignant teratomas compared with the extra-gonadal (90% vs. 10% in male; 83% vs. 17% in female). For the male, the total of the gonadal and the extra-gonadal were all significantly decreased from 1973 to 2014 (*p* < 0.05). For the female, there were no significant trends. As for prevalence, incidence, and frequency, there were two separate peaks of malignant teratomas. One peak was at under 1 year old, which was composed of the extra-gonadal tumor; the other peak was at 20–24 for male and 10–34 for female, which was composed of the gonadal tumor. This separation of the gonadal and extra-gonadal showed a significant difference (*p* < 0.05). As for the prognosis, the extra-gonadal tumor showed significantly lower survival rates than the gonadal (p < 0.05). In the short term, the survival rate of the chemotherapy group was higher than the supportive care group. However, in the long term, the survival rate of the chemotherapy group was lower than the supportive care group.

**Conclusion:**

The gonadal and extra-gonadal malignant teratomas show lots of differences. Chemotherapy might not help improve survival rates.

**Electronic supplementary material:**

The online version of this article (10.1186/s12885-019-5598-0) contains supplementary material, which is available to authorized users.

## Background

A malignant teratoma, or immature teratoma, is a type of germ cell tumor which consists of tissue derived from the three germ layers—ectoderm, mesoderm, and endoderm. However, it is very rare and uncommon. This tumor has had a variety of other names throughout history, such as solid teratoma, teratoblastoma, teratocarcinoma, and embryonal teratoma [1]. In contrast to the mature or benign teratoma, these tumors are large, encapsulated masses with rich solid components, composed of immature elements and neuroepithelial components [2, 3]. The etiology of a malignant tumor was considered as multifactorial, including chromosomal abnormalities and abnormalities in early embryonic development [4, 5].

There is very little research on the malignant teratoma. Only a few pieces of literature have studied it as case reports or case series [6–9], and some other pieces of literature focus on women’s ovarian teratomas, but rarely on men’s. Some pieces of literature thought malignant teratoma affected young women during the first two decades of life, with a median age at 19 years [3, 10, 11]; some literature suggested it could be diagnosed at any age and showed a rapid increase after age 50 [12]. As for the information about its original location, some studies showed the extra-gonadal teratoma was rare compared with the gonadal teratoma. However, there is little information about the differences between them [13–15]. Treatments of malignant teratoma include both surgery and chemotherapy. As far as the effects of chemotherapy on this disease are concerned, most of the studies thought chemotherapy would help to improve the prognosis [16–19], while few studies thought it might not help [20].

In order to learn the differences between the gonadal and extra-gonadal malignant teratomas, we studied them for both male and female, including the original locations, grades, extensions, trends in incidence, estimated prevalence, incidences, frequency, and multiple survival rates using thousands of cases in National Cancer Institute’s Surveillance, Epidemiology, and End Results (SEER) database from 1973 to 2014. In addition to this, we observed the effects of chemotherapy by comparing the survival rates of different groups.

## Methods

### Data

The data used in our research was provided by the National Cancer Institute’s Surveillance, Epidemiology, and End Results (SEER) database [21]. The National Cancer Institute runs the SEER program as a source of cancer information, for example, incidence, prevalence, survival, second incidence of cancer, etc. The registry used in our study was the SEER 9 registries Custom Data (with additional treatment fields), Nov 2016 Submission (1973–2014) < Katrina/Rita Population Adjustment> − Linked to County Attributes - Total United States, 1969–2015 Counties, National Cancer Institute, DCCPS (Division of Cancer Control and Population Sciences), Surveillance Research Program, released April 2017, based on the November 2016 submission.

### Patient selection

Patients up to 85+ years old with a malignant teratoma diagnosed in the period of 1973 to 2014 (3799 male and 1832 female) were selected through SEER. Only the first matching record for each individual was included, in order to exclude second malignancy in those patients with multiple primary tumors.

### Cancer subtypes

To analyze the different locations of malignant teratoma [5], we used the International Classification of Disease for Oncology 3rd Edition (ICD-O-3) [22] (9080, 9082, 9083 and 9102) and the International Classification of Childhood Cancer (ICCC), 3rd Edition [23] (X(a.2) with behavior malignant, X(b.2) and X(c.2)) to classify and evaluate malignant teratomas. In this analysis, we included only teratomas coded as malignant in the SEER database.

### Statistical analysis

Based on the original locations of a malignant teratoma, it was divided into two classes: gonadal and extra-gonadal. For the extensions of the tumor, the malignant teratoma was divided into three classifications: localized, regional, and distant. The tumor grade in our study was used according to SEER coding system for solid tumor (four-grade system). The four-grade system describes the tumor as a Grade I, also called well-differentiated; b. Grade II; also called moderately differentiated; c. Grade III; also called poorly differentiated; d. Grade IV; also called undifferentiated or anaplastic. The data analyzed and presentations were made by SEER*Stat (version 8.3.4), Microsoft Excel (version 2010), and PerkinElmer TIBCO Spotfire (version 7.11.0).

The trends were calculated by the weighted least squares method [24]. The rates were presented as number per 1,000,000, and the age-adjusted models were according to the 2000 US Standard Population (19 age groups - Census P25–1130) standard. Confidence intervals (CIs) were considered as 95% for rates (Tiwari mod) and trends. Percent changes were calculated by using one year for each end. The average annual percentage changes (APCs) were also calculated by using the weighted least squares method. The APC was considered significant if the confidence interval did not include zero (*p* < 0.05).

The estimated prevalence, incidence rates, and frequency were also calculated. The estimated prevalence was calculated according to the January 1, 2014, 39-Year Limited Duration Prevalence. Populations were estimated by averaging 2013 and 2014 populations. The estimated prevalence and incidence rates were presented as number per 1,000,000 persons, and the age-adjusted models were according to the 2000 US Standard Population (19 age groups - Census P25–1130) standard. The rate ratio was calculated to compare the difference between gonadal and extra-gonadal groups (*p* < 0.05).

Survival rates were calculated by the five-year observed survival rates, expected rates and relative survival rates with age adjustment [25, 26]. The relative survival rate was taken by the observed-to-expected survival rates. Kaplan-Meier method was used and the Ederer II method was used for cumulating the expected. These rates were reported as percentages. The ages of patients were standardized to the International Cancer Survival Standard 1 - Ages 15+ in SEER. The expected rates were based on data from the National Center for Health Statistics and differences in distributions of age, sex, race, and year of diagnosis were considered.

## Results

**Fig. 1:**
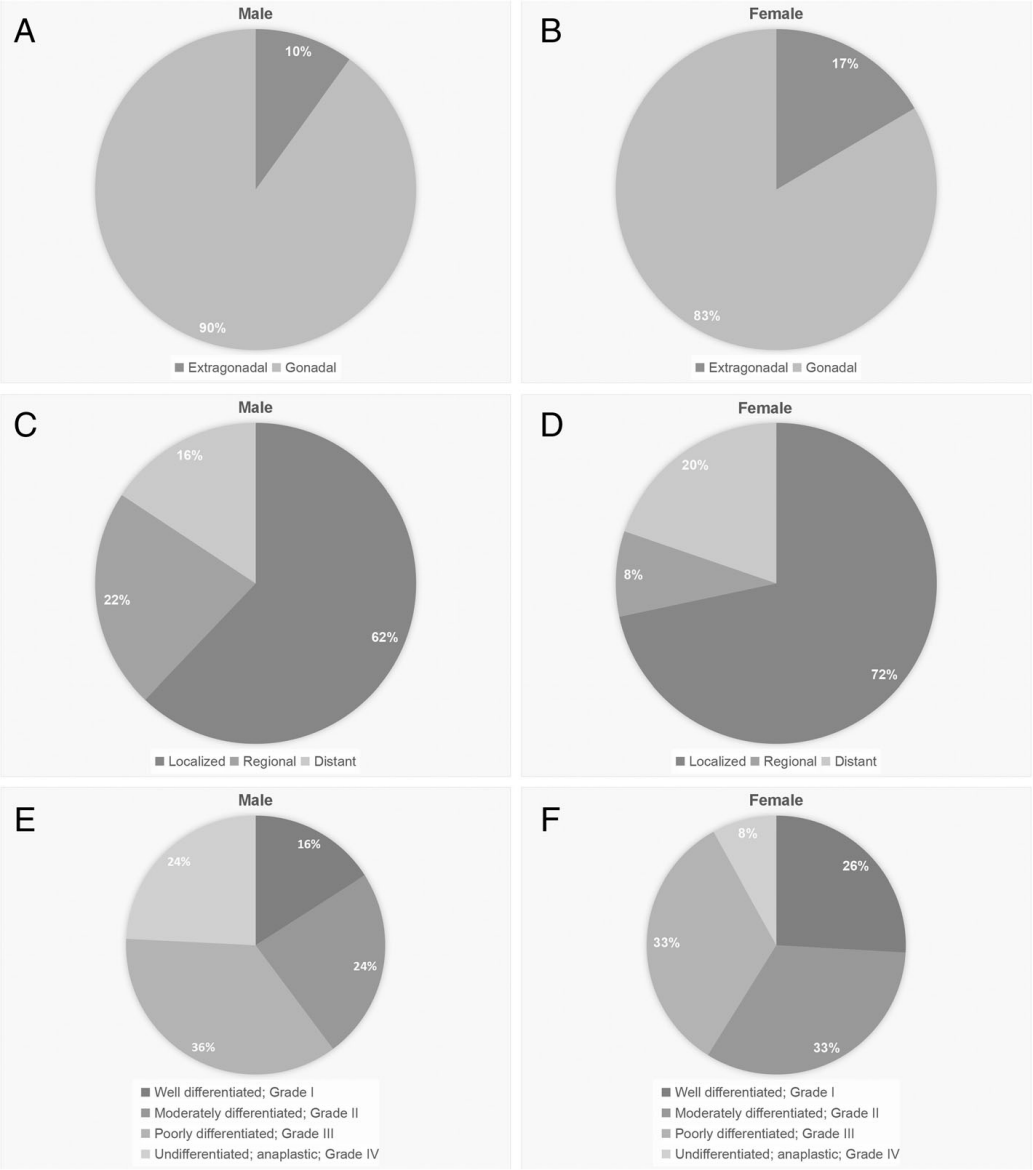
The percentage of malignant teratoma divided by location, extension, and grade in male and female in the SEER registry, 1973–2014. The percentage of malignant teratoma in male and female divided by location (**a**, **b**). The percentage of malignant teratoma in male and female divided by extension (**c**, **d**). The percentage of malignant teratoma in male and female divided by grade (**e**, **f**)

**Fig. 2:**
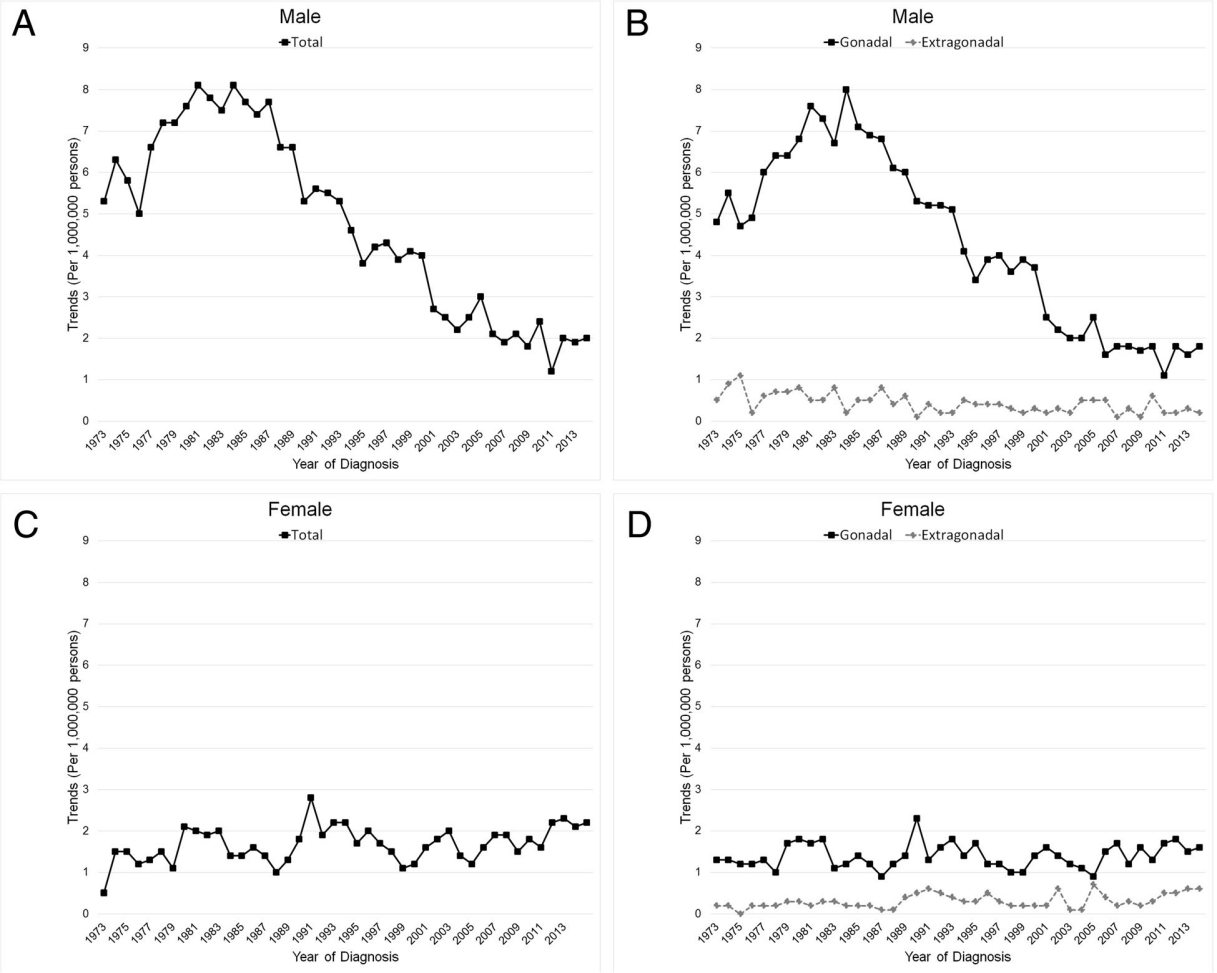
The trends in incidence with age adjustment of malignant teratomas. Trends in incidence of malignant teratoma by year of diagnosis in male (**a**) and divided into the gonadal group and the extragonadal group (**b**). Trends in incidence of malignant teratomas by year of diagnosis in female (**c**) and divided into the gonadal group and the extragonadal group (**d**)

From 1973 to 2014, there were 3799 male and 1832 female malignant teratoma records found in the SEER registries. First, we separated the malignant teratomas by their original locations (Fig. 1A, B). The results showed a majority percentage of the gonadal tumor. Also, we observed the extensions of malignant teratoma and the percentage of grades showed in the (Fig. 1C, D, E, F). In male age-adjusted trends (Fig. 2A, B), we observed a significantly decreased trend of malignant teratoma (APC = -3.7; 95% CI (− 4.3, − 3); *p* < 0.05), dropping from 5.3 per 1,000,000 persons at the year 1973 to 2 at 2014. However, by cutting at 1985, we can observe an increased trend before 1985 (APC = 2.9; 95% CI (1.3, 4.6); *p* < 0.05) and a decreased trend after 1985 (APC = -3.4; 95% CI (− 3.9, − 3); *p* < 0.05). By dividing the tumor into two groups, gonadal and extra-gonadal, we observed that the trends were separated. The extra-gonadal trend was much lower than the gonadal one (from 0.5 in 1973 to 0.2 in 2014, APC = -2.4; 95% CI (− 3.5, − 1.3); *p* < 0.05). In female age-adjusted trends (Fig. 2C, D), there were no significant trends (from 0.5 in 1973 to 2.2 in 2014, APC =0.5; 95% CI (− 0.1, 1.1); *p* > 0.05). The extra-gonadal trend was much lower than the gonadal one (from 0.2 in 1973 to 0.6 in 2014).

**Fig. 3:**
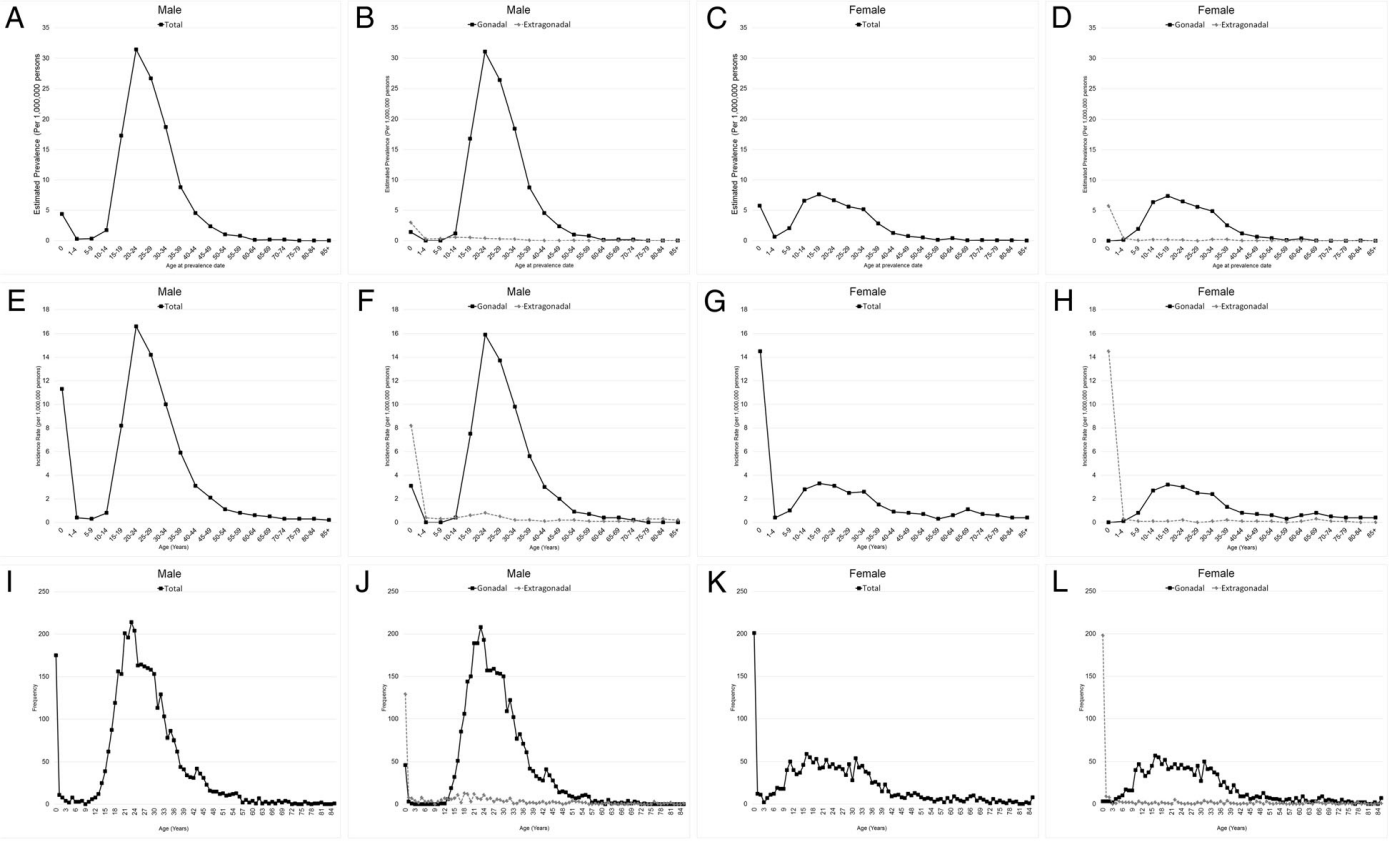
The estimated prevalence, incidences, and frequencies with age adjustment of malignant teratomas. The estimated prevalence, incidences, and frequencies of malignant teratoma by year of diagnosis in male (**a**, **e**, **i**), and divided into the gonadal group and the extragonadal group (**b**, **f**, **j**). The estimated prevalence, incidences, and frequencies of malignant teratoma by year of diagnosis in female (**c**, **g**, **k**), and divided into the gonadal group and the extragonadal group (**d**, **h**, **l**)

Then we calculated the estimated prevalence with age adjustment (Fig. 3 A, B, C, D). We observed two peaks in both genders. For the male, one peak was under 1 year old (4.37 per 1,000,000 persons), and the other was at 20–24 (31.42). The peak under 1 year old was mainly composed of the extra-gonadal tumor (2.96), and the peak at 20–24 was mainly composed of the gonadal tumor (31.05). For the female, one peak was under 1 year old (5.73), and the other peak was at 10–34 (7.6). The peak under 1 year old was mainly due to the extra-gonadal tumor (5.73), and the peak at 10–34 years old was mainly caused by the gonadal tumor (7.4). To study further, we analyzed the incidence rate with age adjustment (Fig. 3 E, F, G, H). For the male, one peak was under 1 year old (11.3 per 1,000,000 persons), and the other peak was at 20–24 (16.6). The peak under 1 year old was mainly composed of the extra-gonadal tumor (8.2), and at 20–24 was mainly gonadal (15.9). We compared these two groups by rate ratio (RR). The significant differences between two groups were at 0–4 years old, and at 10–74 (*p* < 0.05), including two peaks of under 1 year old (RR = 2.6250; 95% CI (1.6170, 4.3934); *p* < 0.01) and at 20–24 (RR = 0.0472; 95% CI (0.0316, 0.0681); *p* < 0.01). For the female, one peak was under 1 year old (14.5), and the other peak was at 10–34 (3.3**)**. The peak under 1 year old was mainly composed of the extra-gonadal tumor (14.5), and the peak at 10–34 was mainly composed of the gonadal tumor (3.2). The significant differences between the two groups were under 1 year old and at 05–69 years old (*p* < 0.05), including the peak at 15–19 (RR = 0.0246, 95% CI (0.0050, 0.0736); *p* < 0.01). Also, we observed the frequency by age during the 1-year interval (Fig. 3 I, J, K, L).

**Fig. 4:**
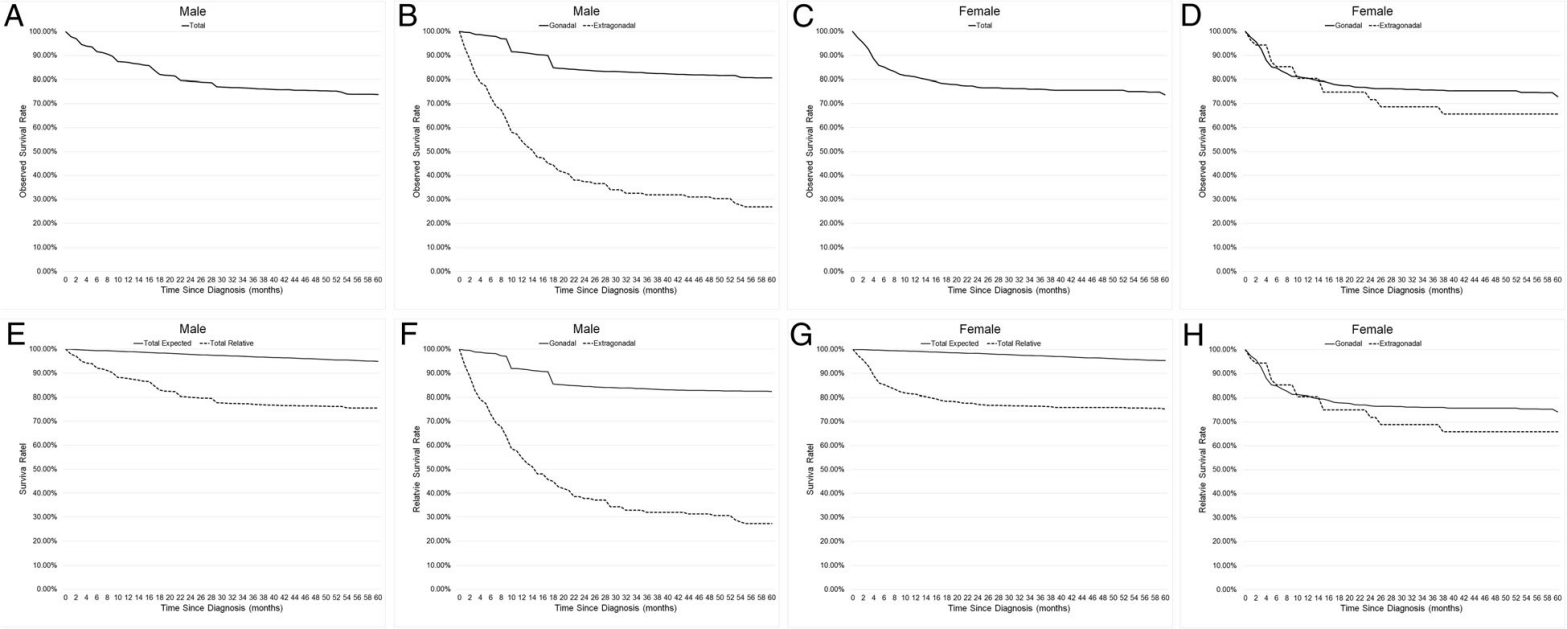
The five-year observed survival rates, expected survival rates, and relative survival rates with age adjustment of malignant teratomas. The observed survival rates of malignant teratomas in male and female (**a**, **c**), and divided into the gonadal group and the extragonadal group (**b**, **d**). The expected survival rates, and relative survival rates of malignant teratomas in male and female (**e**, **g**). The relative survival rates of malignant teratomas divided into the gonadal group and the extragonadal group in male and female (**f**, **h**)

The perinatal malignant teratoma (0 years) is a special subgroup of patients. In the male, the estimated prevalence (4.37 per 1,000,000 persons), incidence rate (11.3), and frequency (175) in the perinatal group were mainly caused by the extra-gonadal (2.96, 8.2, 129) and a small part of the gonadal. In the female, the estimated prevalence (5.73), incidence rate (14.5), and frequency (201) in the perinatal subgroup were mostly the extra-gonadal. The results indicated two pieces of information: 1) The majority of extra-gonadal tumors occurred in the perinatal subgroup. 2) Unlike females, a very small part of the gonadal tumor in males also occurred in the perinatal subgroup. We also ran survival rates of the perinatal subgroup, and the results of the perinatal female children are similar to the results of the extra-gonadal tumor (Fig. 4D, H) because in females the perinatal subgroup is almost entirely made up of extra-gonadal tumors. In males, the five-year observed survival rates of perinatal extra-gonadal tumors were 71.80%, and of the perinatal gonadal tumor were 91.10% (Additional file 1: Figure S1, Additional file 2: Figure S2).

We observed survival rates of gonadal and extra-gonadal tumors, with age adjustment (Fig. 4**)**. In males, the five-year observed survival rate was 73.9%. By separation, the observed survival rate of extra-gonadal tumors (26.8%) was lower than the gonadal (80.6%). Compared with the expected rate (94.9%), the relative rate was lower (75.5%). Compared with the gonadal tumor (82.3%), the five-year relative survival rate of the extra-gonadal tumor (27.3%) was significantly much lower (Z-Value = − 6.021, *p* < 0.01). In females, the observed survival rate was 73.7%. The five-year observed survival rate of the extra-gonadal tumor (65.6%) was lower than the gonadal (72.9%). Compared with the expected rate (95.3%), the relative rate was lower (75.2%). Compared with the gonadal tumor (74.2%), the relative survival rate of the extra-gonadal tumor (65.9%) was significantly much lower (Z-Value = − 3.604, *p* < 0.01).

**Fig. 5:**
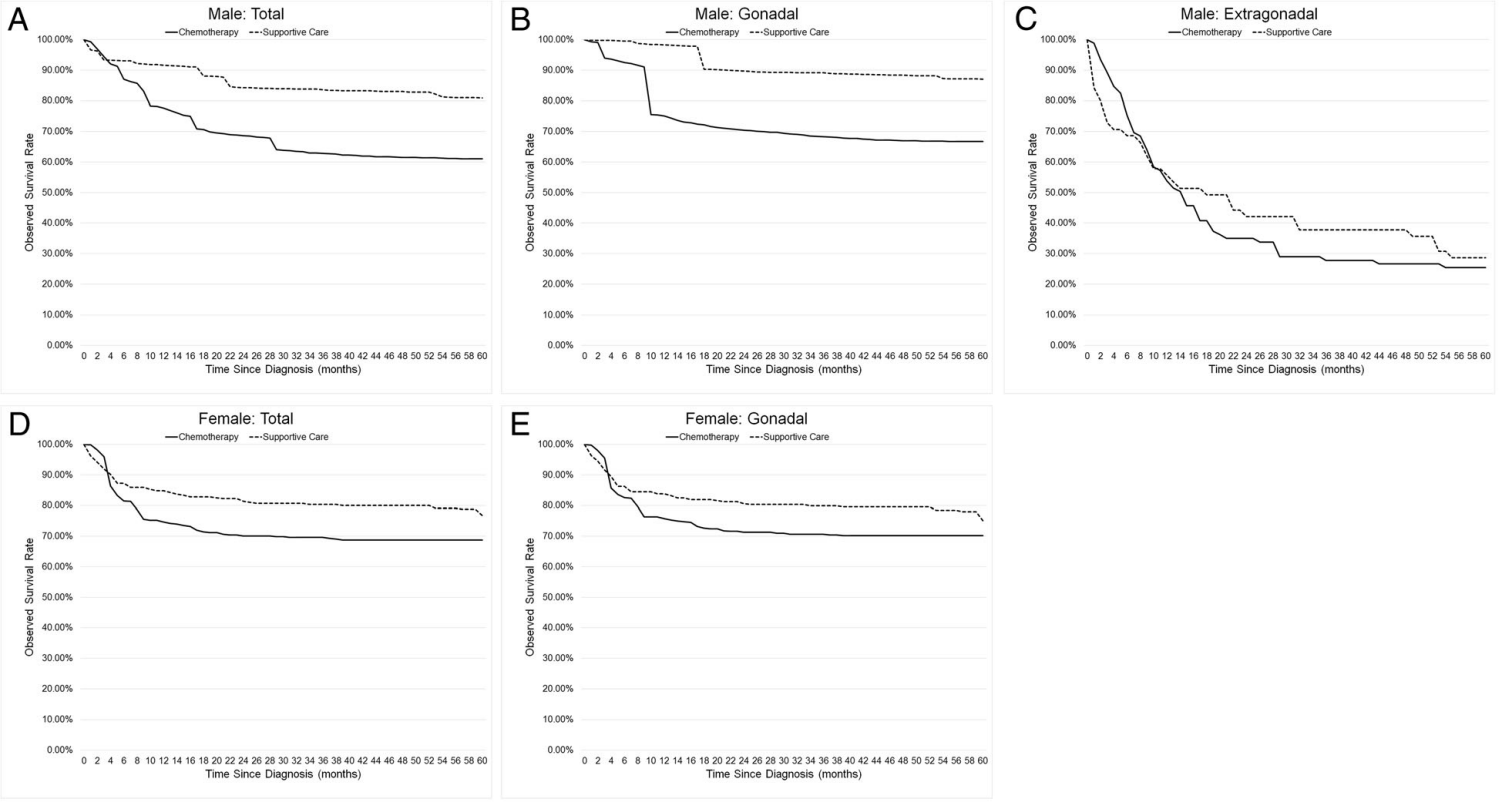
The five-year observed survival rates with age adjustment of malignant teratomas divided by chemotherapy and supportive care groups. The observed survival rates of malignant teratomas divided by chemotherapy and supportive care groups in male and female (**a**, **d**). The observed survival rates of gonadal malignant teratomas divided by chemotherapy and supportive care groups in male and female (**b**, **e**). The observed survival rates of extragonadal malignant teratomas divided by chemotherapy and supportive care groups in male (**c**)

To study the effects of chemotherapy, we observed the survival rate with age adjustment (Fig. 5). In males, during the first three months, the observed survival rate of the chemotherapy group was higher. After that, the observed survival rate of the chemotherapy group (61.1%) was lower than the supportive care group (80.9%). In gonadal tumors, the rate of the chemotherapy group (66.8%) was lower than the supportive care group (87.7%). In extra-gonadal tumors, the rate of the chemotherapy group was higher than the supportive group in the first ten months of follow-up, whereas it was lower afterwards until 60 months. In females, in the first three months, the observed survival rate of the chemotherapy group was higher. After that, the observed survival rates of the chemotherapy group (68.8% in total, 70.2% in gonadal) were lower than the supportive care group (76.8% in total, 75.1% in gonadal).

## Discussion

Malignant teratoma can be classified into two groups with respect to its location: the gonadal and extra-gonadal tumor. The gonadal type of malignant teratoma seen in the gonads and possibly in the posterior abdominal wall is derived from germ cells by a process of parthenogenesis. The extra-gonadal tumor seen in the sacrococcygeal region, the head, and the chest is related to sequestration of cells of the blastula before differential blocking of the genome has occurred [5]. Malignant teratoma is an uncommon tumor, most of which are case reports or case series on female ovaries [6–9].

For the gonadal malignant teratoma, the studies were limited to female ovaries. Deodhar [10] et al. observed 28 cases of malignant teratoma on an ovary. They showed that the median age for the cases was 19 years, and 1 patient died at 7 months. Norris et al. [11] observed 58 cases of malignant teratoma of the ovary. They showed the actual survival rate was 63% at 5 years. O Solheim et al. [12] observed 351 patients with malignant ovarian germ cell tumors. They showed that the malignant teratoma could be diagnosed at any age with a rapid increase after age 50 years. For the extra-gonadal malignant teratoma, the studies were rare. Thurkow et al. [13] observed one extremely rare case of malignant teratoma of the neck, with mature and immature metastatic lesions in the lungs, in an immature fetus. Bauman and Nerlich [14] observed one case of a metastatic cervical teratoma of the fetus. Shoenfield et al. [15] wrote an article review in 2009, with only six reports of cervical malignant teratoma in the literature so far. However, there is almost no research about the comparisons of gonadal and extra-gonadal malignant teratomas.

In our studies, the gonadal took up a majority percentage of malignant teratoma compared with the extra-gonadal (90% vs. 10% in male; 83% vs. 17% in female), which was well consistent with literature. For male trends, the total, the gonadal, and the extra-gonadal were all significantly decreased from 1973 to 2014. For females, there were no significant trends. As for prevalence, incidence, and frequency, there were two separate peaks of malignant teratoma. One peak was under 1 year old, which was composed of the extra-gonadal tumor; the other peak was at 20–24 in males or 10–34 in females, which was composed of the gonadal tumor. This separation of the gonadal and extra-gonadal showed a significant difference. As for the prognosis, the extra-gonadal tumor showed significantly lower survival rates than the gonadal. These lower survival rates in extra-gonadal malignant teratoma might be caused by the fetus with very low immunity, surgery, and chemotherapy treatments since our study found the extra-gonadal tumor’s predilection age was under 1 year old.

Treatments of malignant teratoma include surgery and chemotherapy. As far as the effects of chemotherapy on this disease are concerned, most of studies thought that chemotherapy helps improve the prognosis [16–19] because it might determine tissue maturation, giving it an appearance more typical of a mature cell type which remains stable for a long period of time [18, 19]. However, in other studies, like in Pashankar’s research, postoperative chemotherapy did not decrease relapses in the pediatric cohort [20], thus the effect of chemotherapy showed a conversed result in the short- and long-term survival rates. In the short term, the survival rate of the chemotherapy group was higher than the supportive care group. However, in the long term, the survival rate of the chemotherapy group was lower than the supportive care group. By our results, it might explain the controversy of different literatures.

However, there are still some limitations to our study. First, the data we collected was only from the National Cancer Institute’s Surveillance, Epidemiology, and End Results (SEER) database, which covers about 9% of American cancer citizens. However, there are still 91% of cancer citizens out of the picture, which may be different from the 9% one. Second, our study lacks information on the nature of the presence of the heterologous component, for example, the information of malignant transformation. Thirdly, our study lacks information on mixed components, like the information about yolk sac tumors or embryonal carcinomas. Moreover, the data is limited to the United States, without information from other Western countries, Asia, or the rest of the world. Finally, the data of our study was from 1973 to 2014, without considering the influence of the development in diagnosis and treatments over the years.

## Conclusion

Significant trends of malignant teratoma in males increased from 1973 to 1985 and decreased till 2014. There were two peaks of malignant teratoma, the first peak being under 1 year old, which was mostly caused by the extra-gonadal tumor, and the second peak was at middle age, which was mostly caused by the gonadal tumor. The treatment of chemotherapy in malignant teratoma might help increase the survival rate in a short period of time, while it might not help in the long run.

## Additional files

Additional file 1: **Figure S1.** The five-year observed survival rates of malignant teratomas in the perinatal subgroup of male infants. (JPG 224 kb)

Additional file 2: **Figure S2.** The five-year observed survival rates of malignant teratomas in the perinatal subgroup of male infants with the gonadal group and the extragonadal group. (JPG 235 kb)

## Abbreviations

APCs: Annual percentage changes
CIs: Confidence intervals
DCCPS: Division of cancer control and population sciences
ICCC: International classification of childhood cancer
ICD-O-3: International classification of disease for oncology 3rd edition
RR: Rate ratio
SEER: Surveillance, epidemiology, and end results
