# Supplementary figures and images for: The differences between gonadal and extra-gonadal malignant teratomas in both genders and the effects of chemotherapy
Source: BMC Cancer. 2019 Apr 30;19:408. doi: 10.1186/s12885-019-5598-0 (PMC6492338; doi:10.1186/s12885-019-5598-0)

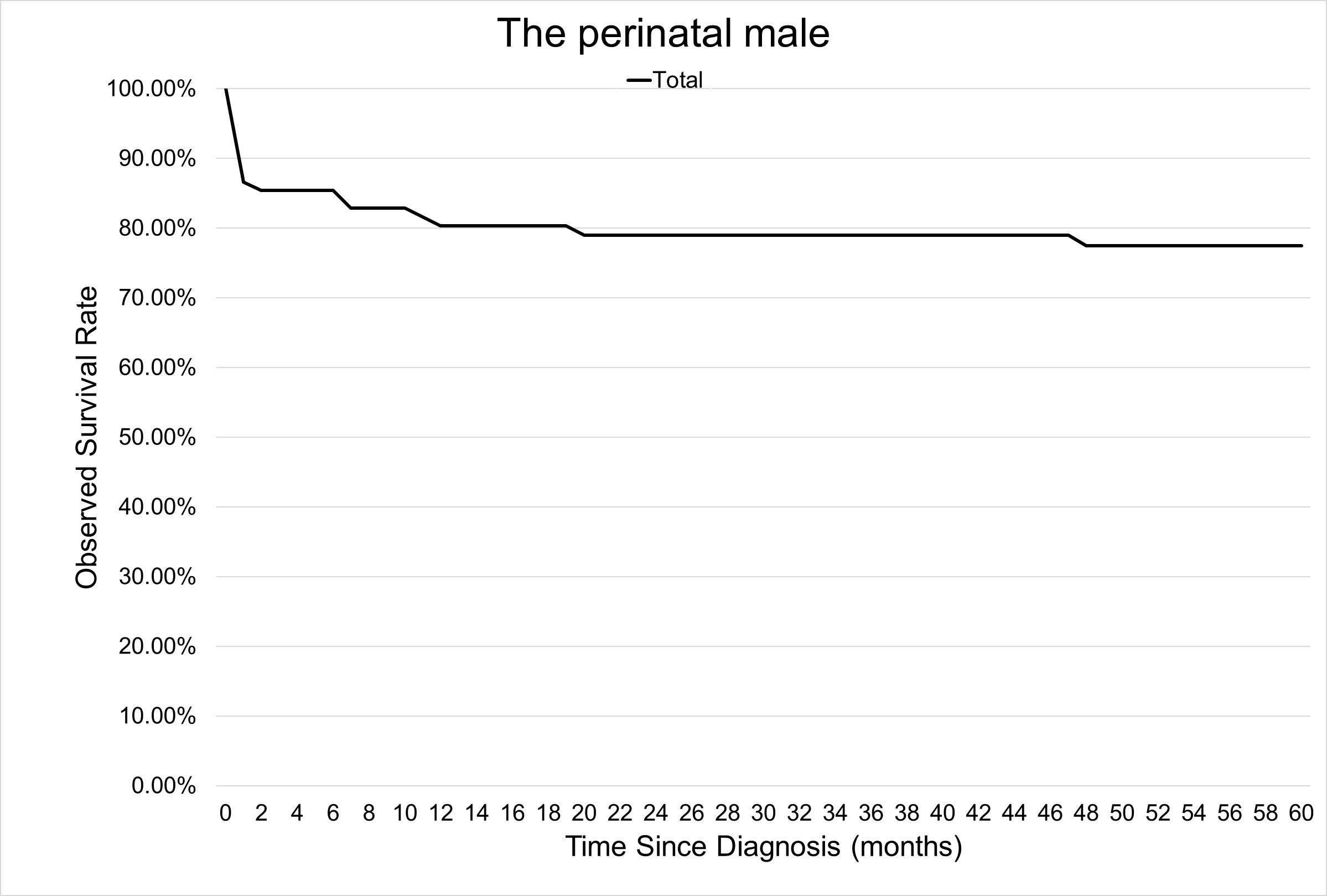

Supplement: Supplementary file 1 — Figure S1. The five-year observed survival rates of malignant teratomas in the perinatal subgroup of male infants. (JPG 224 kb) [file 12885_2019_5598_MOESM1_ESM.jpg]

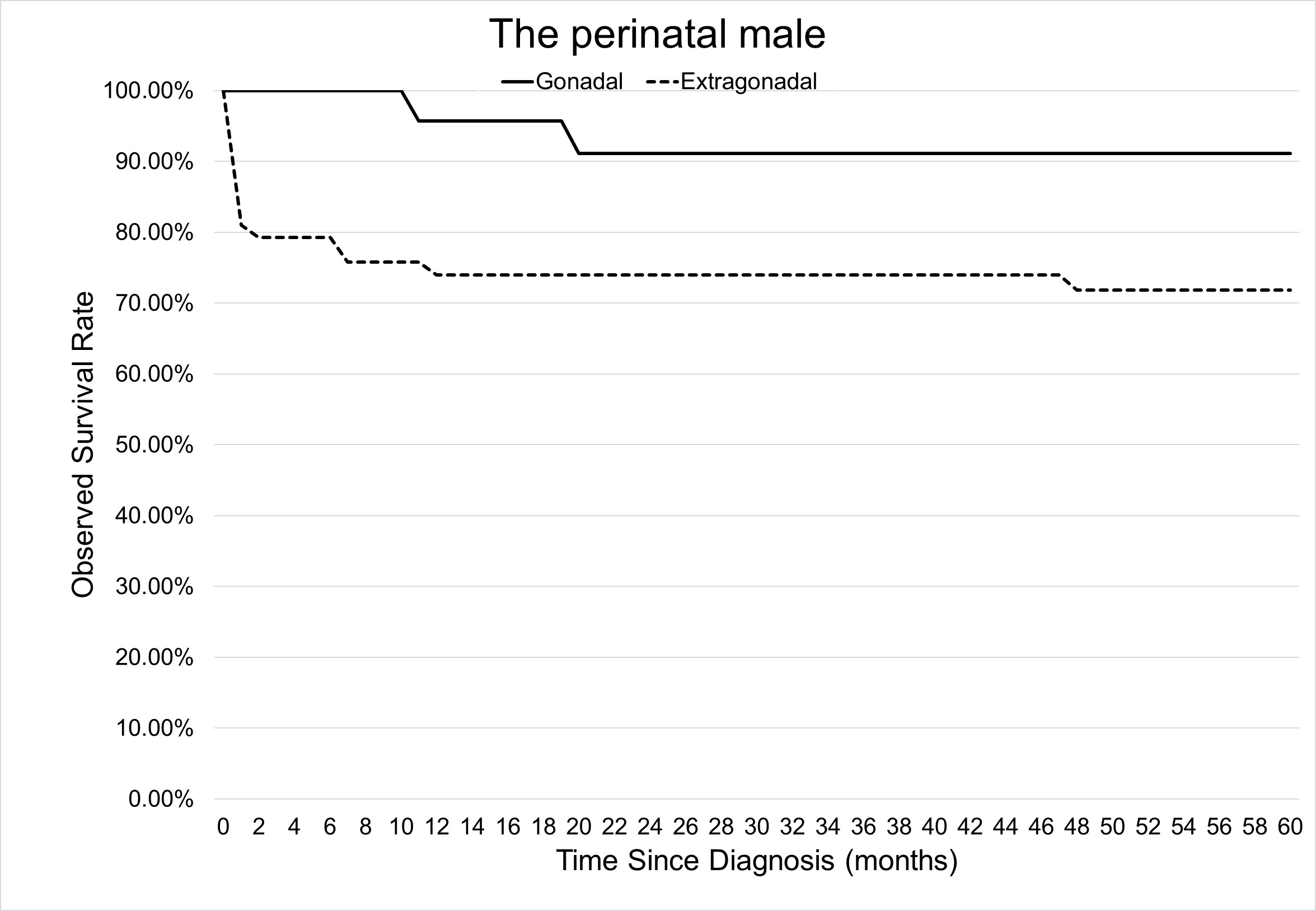

Supplement: Supplementary file 2 — Figure S2. The five-year observed survival rates of malignant teratomas in the perinatal subgroup of male infants with the gonadal group and the extragonadal group. (JPG 235 kb) [file 12885_2019_5598_MOESM2_ESM.jpg]
